# Increasing the production of the bioactive compounds in medicinal mushrooms: an omics perspective

**DOI:** 10.1186/s12934-022-02013-x

**Published:** 2023-01-16

**Authors:** Nooshin Arshadi, Hoda Nouri, Hamid Moghimi

**Affiliations:** Department of Microbial Biotechnology, School of Biology, College of Science, University of Tehran, Tehran, Iran

**Keywords:** Bioactive compound, Medicinal mushroom, Metabolic engineering, Omics study, Systems biology, Transcriptomics

## Abstract

Macroscopic fungi, mainly higher basidiomycetes and some ascomycetes, are considered medicinal mushrooms and have long been used in different areas due to their pharmaceutically/nutritionally valuable bioactive compounds. However, the low production of these bioactive metabolites considerably limits the utilization of medicinal mushrooms both in commerce and clinical trials. As a result, many attempts, ranging from conventional methods to novel approaches, have been made to improve their production. The novel strategies include conducting omics investigations, constructing genome-scale metabolic models, and metabolic engineering. So far, genomics and the combined use of different omics studies are the most utilized omics analyses in medicinal mushroom research (both with 31% contribution), while metabolomics (with 4% contribution) is the least. This article is the first attempt for reviewing omics investigations in medicinal mushrooms with the ultimate aim of bioactive compound overproduction. In this regard, the role of these studies and systems biology in elucidating biosynthetic pathways of bioactive compounds and their contribution to metabolic engineering will be highlighted. Also, limitations of omics investigations and strategies for overcoming them will be provided in order to facilitate the overproduction of valuable bioactive metabolites in these valuable organisms.

## Background

The application of mushrooms for medicinal purposes has a very long history [1]. Macroscopic fungi, mainly higher Basidiomycetes and some Ascomycetes*,* are considered medicinal mushrooms and can prevent, alleviate or cure several diseases and balance a healthy diet in the form of powders or extracts [2]. Many higher Basidiomycetes contain high/low molecular weight compounds, such as polysaccharides [3], lectins [4], triterpenes [5], statins, phenolic compounds, and antibiotics, in their fruit bodies, cultured mycelia, and cultured broth [6,7]. According to previous studies, some medicinal properties detected in mushrooms are as follows: antioxidant, antiviral, antifungal, antibacterial [4], antiobesity [8], cardiovascular protective [9], neuroprotective [10], immunomodulating, antitumor [3], hepatoprotective, cholesterol-lowering [11], antidiabetic [12], neuroregenerative, radical scavenging, and detoxicating activities [2,6,13]. For example, *G. lucidum*, a medicinal mushroom that possesses therapeutic activities such as antitumor, antioxidant, and immunomodulatory effects, is used for postponing aging, improving health, preventing and curing illnesses such as hypertension, gastric cancer, hepatitis, bronchitis as well as minor disorders including insomnia. In fact, it is possible to manufacture several valuable Ganoderma-based products, including soft capsules, injections, tablets, and drinks, by utilizing their spores and basidiocarps [14]. Thus, medicinal mushrooms are important for modern medicine and can be used as a new class of drugs known as “Mushroom Pharmaceuticals” to support a good quality of life and prevent illnesses such as immune system diseases [2,15].

From 1990 to 2020, global mushroom production has raised 13.8-fold to 42.8 million tons [16]. This global industry, which is consisted of edible, medicinal, and wild mushrooms, was approximated to be about $63 billion in 2013, with China being the leading producer of cultivated, edible mushrooms. 54% of this global industry is designated to cultivated, edible mushrooms and was around $34.1 billion in 2015 [17,18]. However, as more increase in edible mushroom consumption is anticipated in upcoming years, annual sales of this component of the world mushroom industry will grow from $34 to $60 billion [19] and their market will reach 24.05 million tons by 2028 [20]. Moreover, the remaining components of the global industry, namely medicinal mushrooms and wild mushrooms, represented 38% ($24 billion) and 8% ($5 billion) of the total value, respectively [17]. 85% of total mushroom production in the world is allocated to five fungal genera, i.e., *Lentinula* (the main genus) having about 22%, *Pleurotus* (mainly *P. ostreatus*, besides *P. eryngii*, *P. djamor, P. pulmonarius,* and *P. citrinopileatus*) with roughly 19% and *Auricularia* with approximately 17% of the world’s production. Next are *Agaricus* (mostly *A. bisporus* and considerably lower *A. brasiliensis* amounts) and *Flammulina*, the fourth and fifth most cultivated mushrooms, with 15% and 11% of the total amount, respectively [21]. Some other cultivated mushroom species are *G. lucidum, V. volvacea, H. erinaceus, G. frondosa*, and *T. versicolor*, which are desired edible and medicinal species in many regions of the globe [1]. Based on the most recent estimations, the market size of *G. lucidum* products is worth over US $2.5 billion [14,22]. Furthermore, It was estimated that the annual production of *V.volvacea* is 330,000 tons in China [23]. *Antrodia cinnamomea* and *Cordyceps militaris* are two other examples of medicinal mushrooms. According to estimations, products derived from *A. cinnamomea*, such as health foods and raw fruiting bodies, have a total market value of more than US$ 100 million annually [24], and the annual sale of *C. militaris* was evaluated to be about 3 billion RMB in China [25]. Although the current reach for other medicinal mushrooms may not be extensive at a global level, creating awareness about these mushrooms and their benefits will eventually increase their market potential.

Several examples of medicinal mushrooms (mainly those related to our review), their bioactive substances, medicinal properties, and applications are summarized in Table 1. In addition to the utilization of mushrooms as “Mushroom Pharmaceuticals,” they can be used as dietary foods, dietary supplement products, additive and ingredient replacers (such as meat substitutes) [26], cosmeceuticals [2,15], and analgesics [27]. Furthermore, as they possess insecticidal, fungicidal, nematocidal, antiphytoviral, bactericidal, and herbicidal effects, they can be utilized as natural biocontrol agents for plant protection [2,28]. There is an increasing demand for mushrooms due to all of the applications mentioned above as well as the nutritional value and pharmaceutical properties of their bioactive compounds. However, the low production of their bioactive compounds can be a bottleneck for clinical trials and commercial applications [29]. For example, improvements in the production of GA-T (a bioactive substance in *G. lucidum)* are needed to decrease production expenses and fulfill the demands in large-scale, commercial, and clinical trial fields [30]. Hence, many efforts have been made to increase the production yield of bioactive compounds in medicinal mushrooms via different methods such as optimizing the growth conditions (medium components and cultivation conditions) [31,32], signal transduction induction by inducers [5], and applying heat stress [33].

**Table 1:** Examples of medicinal mushrooms, their bioactive substances, medicinal properties, and applications

Mushroom	Bioactive compounds	Medicinal properties	Other applications	References
*Pleurotus spp. *(oyster mushrooms)	Lovastatin, ergothioneine, lectins, triterpenoids, peptides, polysaccharides (such as Pleuran)	Antitumor, immunomodulatory, anti-inflammatory, and antioxidant activities	•Lowering cholesterol levels and blood pressure if added to animal diet •Pleuran: effective for preventing recurrent respiratory tract infections	[1,35,36]
*Lentinula edodes *(shiitake)	Lentinan, eritadenine (lentinacin), lentin, polysaccharide KS-2, lentinamycin, considerable amounts of ergosterol, and γ-aminobutyric acid	Antitumor, cholesterol-lowering, antimicrobial and antiviral activities	•Lentinan: suppressing multiplication of leukemic cells •Eritadenine: considerably decreasing cholesterol and triglyceride •KS-2 polysaccharides: active against Ehrlich and Sarcoma-180 tumors •Ethanol extracts derived from fruiting bodies: reducing CH72 cells multiplication •Lentinamycin, lentin, and lentinan: possessing antimicrobial and antiviral effects	[1,37–44]
*Flammulina velutipes *(enokitake)	Vitamins, amino acids, steroids, phenolic acids, fatty acids Polysaccharides Terpenoids Sterols Agglutinins Proteins Fip-FVE protein and enokipodins Flammulin protein Proflamin glycoprotein(a weakly acidic glycoprotein) effects Ergosterol	Antitumor, acetylcholinesterase inhibitory, and antioxidant activities Antimicrobial Antitumor and antioxidant Antitumor Immunomodulatory and anti-inflammatory properties Antimicrobial activity Activity against different types of cancer cells Antitumor	•Reducing the level of cholesterol in the human body •Lowering the immunological reaction in the event of food allergies •Protecting fish and meat from oxidation as a preservative in the form of extracts and powders	[1,45–51]
*Flammulina filiformis*	Polysaccharides (glucans and heteropolysaccharides), sesquiterpenes, proteins (FIP-fve), flavonoids, glycosides, phenols	Antioxidant, antitumor, and anti-aging effects anti-atherosclerotic thrombosis inhibition and anticancer properties	Possessing potential for utilization in bioethanol production due to its lignin degradation capacity and alcohol dehydrogenase production	[52–61]
*H.erinaceus *(lion’s mane)	Terpenoids (erinacines), volatile aroma compounds, pyrones (erinapyrones A–C), phenols (hericenone A–E), fatty acids, sterols (erinarol, hericerins, hericenes), and non-ribosomal peptides (fumitremorgin C) Primary polysaccharide compounds Diterpenoids (such as cyathane terpenoids, erinacine A to G) Lectins	Anti-aging and anti-dementia properties Antitumor and antioxidant effects Hypoglycemic, antitumor, neuroprotective, and antibacterial properties Potential neuroprotective effects, assisting the biosynthesis of nerve growth factors in cell cultures Hemagglutinating activity, effective on adhesive features of erythrocytes	•Preventing age-associated neurological illnesses (such as Alzheimer’s disease and Parkinson’s disease) •Protecting against cell death due to endoplasmic reticulum stress •Erinacines and hericenones: increasing the synthesis of nerve growth factor (NGF) in cultured astrocytes	[1,10,62–78]
*Dictyophora indusiata*	Polysaccharides, un- saturated fatty acids, flavones, and vitamins, albaflavenone (might have antibacterial activity), dictyophorines A and B (two sesquiterpenes stimulating the production of nerve growth factor), 5-(Hydroxymethyl)-2-furfural (with anti-tyrosinase activity) dictyoquinazols A, B, and C (quinazoline compounds with neuroprotective activity against excitatory neurotoxins)	Lipofuscin resistance, mental tranquilization, cardiovascular protective activity, antibiosis, antioxidant, antitumor, and immunomodulation activities	•Improving immunity •Beneficial to the eyes	[9,79–89]
*A. cinnamomea*	Polysaccharides, antrocamphin, special ergostane triterpenoids (such as antcins C and K), zhankuic acids A, B, and C	Anticancer, antihypertensive and immunomodulatory properties Antioxidant, hepatoprotective, anti-inflammatory, and antihyperlipidemic activities	•Treating various diseases such as abdominal pains, hypertension, hepatitis B, and cancers •Extracts: healing the effects of alcohol intoxication	[90–98]
*C. militaris*	Cordycepin, ergosterol, mannitol, and exopolysaccharides	Antioxidant, anti-inflammatory, anti-microbial, anti-metastatic effects Cordycepin: possessing antibacterial, anti-leukemia, antifungal, immuno-regulative, cytotoxic (against several types of cancer cells), antitumor properties	•Enhancing the immune system •Cordycepin: a candidate for treating cancer, preventing alcohol-induced hepato-toxicity	[99–103]
*Tolypocladium guangdongense *(*C. guangdongensis*)	Cordycepic acid, polysaccharides, adenosine	Longevity-increasing, anti-fatigue, and anti-inflammatory effects	•Applications in functional food and medicine industries	[104,105]
*G. lucidum*	Polysaccharides, sterols, proteins, above 130 triterpenoids, nucleic acids Ganoderic acids with anticancer, anti-platelet aggregation, antiviral, hepatoprotective, hypocholesterolemic, antitumor, anti-aging, anti-metastasis, anti-HIV, and histamine release inhibition activities 1, 3-b-D-glucan; a carcinostatic compound (with high immunological and antitumor activities) beneficial for immunotherapy	Antitumor, immunomodulatory and hypoglycemic activities	•Preventing and treating different diseases such as hypertension, gastric cancer, hepatitis, bronchitis, insomnia, neurasthenia, asthma, hypercholesterolemia, inflammation, and heart disease •Mycelium from submerged liquid culture: as a functional food in Taiwan for improving immunity, boosting health, delaying aging, and protecting the liver	[11,14,29,106–121]
*Phellinus gilvus*	High contents of phenylpropanoids and triterpenoids, but their major active compounds are not completely identified	Anti-inflammatory, immunomodulatory, anti-carcinogenesis, antidiabetic, antioxidative, neuroprotective, hepatoprotective, and antifungal effects	Curing different illnesses such as inflammation, stomachache, and tumors	[122–124]
*V. volvacea*	Essential amino acids, terpenoids, vitamins, steroids, lectins, polypeptides, and phenolic compounds Proteins volvatoxin and flammutoxin and polysaccharides γ-aminobutyric acid	Antitumor activities Decreasing neuronal excitability in every part of the nervous system and regulating muscle tone of the human body in a direct way	Extracts: possessing high antioxidant effects, preventing cardiovascular and neurodegenerative diseases	[1,125–128]

On the other hand, understanding the biosynthetic pathways of bioactive compounds as well as their complex regulation is necessary for achieving improvements in their production [29]. Thus, omics investigations can be novel, powerful, and beneficial tools in this regard. Still, omics approaches have not been adequately exploited for this purpose.

Omic tools, which provide a comprehensive view of cell metabolites, tissues, and organisms, are used to investigate the identification of genes (genomics), mRNA (transcriptomics), metabolites (metabolomics), and protein production (proteomics) under specific environmental conditions or by a particular approach. By utilizing transcriptomic and proteomic methods, it is possible to explain the roles of the fruiting body and vegetative mycelium during the detection of the genes that control the induction or repression of certain metabolic pathways. Moreover, metabolomics studies help determine the metabolites associated with every cellular process and those involved in different culture conditions [34]. To our knowledge, the genome, transcriptome, proteome, and metabolome studies on medicinal mushrooms for increasing the production of pharmaceutical compounds have been rarely reviewed. In fact, up until now 80 articles have conducted omics investigations on medicinal mushrooms with 48.75% of these studies being influential in bioactive compound overproduction. Thus, the present study aims to review for the first time, the omics analyses with the emphasis on improving bioactive substance production. The production of bioactive compounds will be compared before and after exploiting omics-based overproduction strategies and it will be shown that the maximum generated increase can be as high as fourfold. Challenges of omics technologies in medicinal mushroom research and their possible solutions will also be discussed.

## Genomics studies on different medicinal mushrooms

Since genome data makes discovering and analyzing the biosynthesis of bioactive metabolites easier in higher fungi, chances for conducting research and developing their metabolic products can be provided by advancements in genome sequencing [129]. Up until now, genomic information of some edible/medicinal mushrooms including, *A. bisporus* [130], *V. volvacea* [23], *Schizophyllum commune* [131], *F. velutipes* [132], *H. erinaceus* [133]*, G. lucidum* [134], *C. militaris* [138]*, Lignosus rhinocerotis* [135]*, Ganoderma sinense* [139]*,* and *Sanghuangporus sanghuang* [140] has become available and resulted in gaining new insights into various aspects. The results of these genomic analyses are summarized in Table 2. For instance, genome sequencing of the model mushroom *S. commune* provides deeper knowledge of underlying mechanisms of mushroom formation. This knowledge can be helpful in their bioactive compounds production and their application in industry for achieving enzymes and pharmaceuticals.

**Table 2:** Summary of the genomics studies on edible/medicinal mushrooms

Application of the genomics studies	Mushroom	The investigated processes/fields	Additional remarks	References
Uncovering mechanisms and pathways	*A. bisporus*	Acclimatization to a humic-rich ecological niche in the process of plant degradation	Genetic and enzymatic procedures which control the acclimatization were elucidated	[130]
	*V. volvacea*	•Degradation of the cultivating compost containing agricultural waste •Sexual reproduction •Susceptibility to low temperatures at the level of molecules	Many genes encoding enzymes associated with the degradation of cellulose, hemicellulose, and pectin were identified	[23]
	*S. commune*	•Mushroom formation •Wood-degrading machinery and its capability for breaking down lignocellulose	–	[131]
	*C. militaris*	Medicinal compounds synthesis	–	[138]
	*G.sinense*	•Secondary metabolism •Different defense systems	More than thirty gene clusters associated with the biosynthetic pathways of secondary metabolites, as well as numerous genes contributing to their transport and regulation were identified	[139]
	*S. sanghuang*	Secondary metabolites and their synthesis	334 carbohydrate-active enzymes coding, 343 transporters, and 4 velvet family proteins were identified	[140]
	*Tuber melanosporum*	Evolutionary origins and symbiosis	–	[141]
	*L. edodes*	Lignocellulose degradation	101 lignocellulolytic enzymes were determined and gave insights into the mechanism of lignocellulose degradation	[143]
	*P. eryngii*	Lignin and cellulose degradation	The carbohydrate-active enzymes and oxidoreductases in its genome uncovered the mechanisms of cellulose and lignin bioconversion	[144]
	*A. cinnamomea*	•Sexual development •The production of sesquiterpenoids, antrocamphin, ergostanes, antroquinonol, and triterpenoids	DEGs between mycelia and fruiting bodies as well as 242 proteins in different bioactive metabolite pathways were determined	[97]
Identification of genes, enzymes, gene clusters, and proteins	*F. velutipes*	Ethanol production	58 potential enzymes associated with ethanol production were identified	[132]
	*H. erinaceus*	•Lignocellulose degradation •Biosynthesis of bioactive secondary metabolites	341 carbohydrate-active enzymes related to lignocellulose degradation, 447 transcription factors, and gene clusters contributing to bioactive secondary metabolites biosynthesis were identified	[133]
	*G. lucidum*	•Terpenoid synthesis •Triterpene biosynthesis •Wood degradation	Numerous ligninolytic and carbohydrate-active enzymes, as well as the enzymes of the ganoderic acid biosynthetic pathway were determined	[136,137]
	*P. eryngii*	Degradation of lignocellulose	Insights into CAZymes and oxidoreductases was provided	[144]
Discovering/establishing microsatellite markers databases	*H. erinaceus*	Genome-wide microsatellites	A comprehensive microsatellite markers database was established	[133]
	*Ganoderma boninense*	The biology of *G.boninense* and its diversity	Novel polymorphic microsatellite markers were discovered and established	[147]
Uncovering the evolutionary aspects	*Laetiporus sulphureus*	Lignocellulose breakdown	The evolutionary history and roots of the enzymatic toolbox were refined	[142]
	*Coprinopsis cinerea (Coprinus cinereus)*	Evolution of multicellular fungi	Rates of meiotic recombination were indicated to be low in the parts of the genome that remained unchanged over evolutionary time	[145]
	*Fistulina hepatica*	New wood decomposition mechanisms in Agaricales	Changing its lifestyle to a brown-rot lifestyle may be a continuous procedure in this mushroom	[146]
Improving cultivation	*L. edodes*	Cultivation conditions	A rationale for partially replacing wood sawdust with agricultural wastes in cultivation was offered	[143]
Improving the knowledge about the biological properties	*V. volvacea*	Degradation of the cultivating compost containing agricultural waste	The knowledge about the biological properties associated with the degradation of the mentioned cultivating compost was improved	[38]
	*F. velutipes*	Lignocellulose degradation	The high capacity of this mushroom for lignocellulose degradation was revealed	[132]
	*L. rhinocerotis*	Medicinal properties	Information about the genetic basis of medicinal properties was provided	[135]
Providing predictive platforms	*Omphalotus olearius*	Sesquiterpenoid natural products	A prognosticative framework in Basidiomycota for obtaining sesquiterpenoid natural products was offered	[148]
	*L. rhinocerotis*	Secondary metabolite biosynthesis	A platform for the identification of putative bioactive proteins and enzymes contributing to secondary metabolite biosynthesis was provided	[135]

**Fig. 1:**
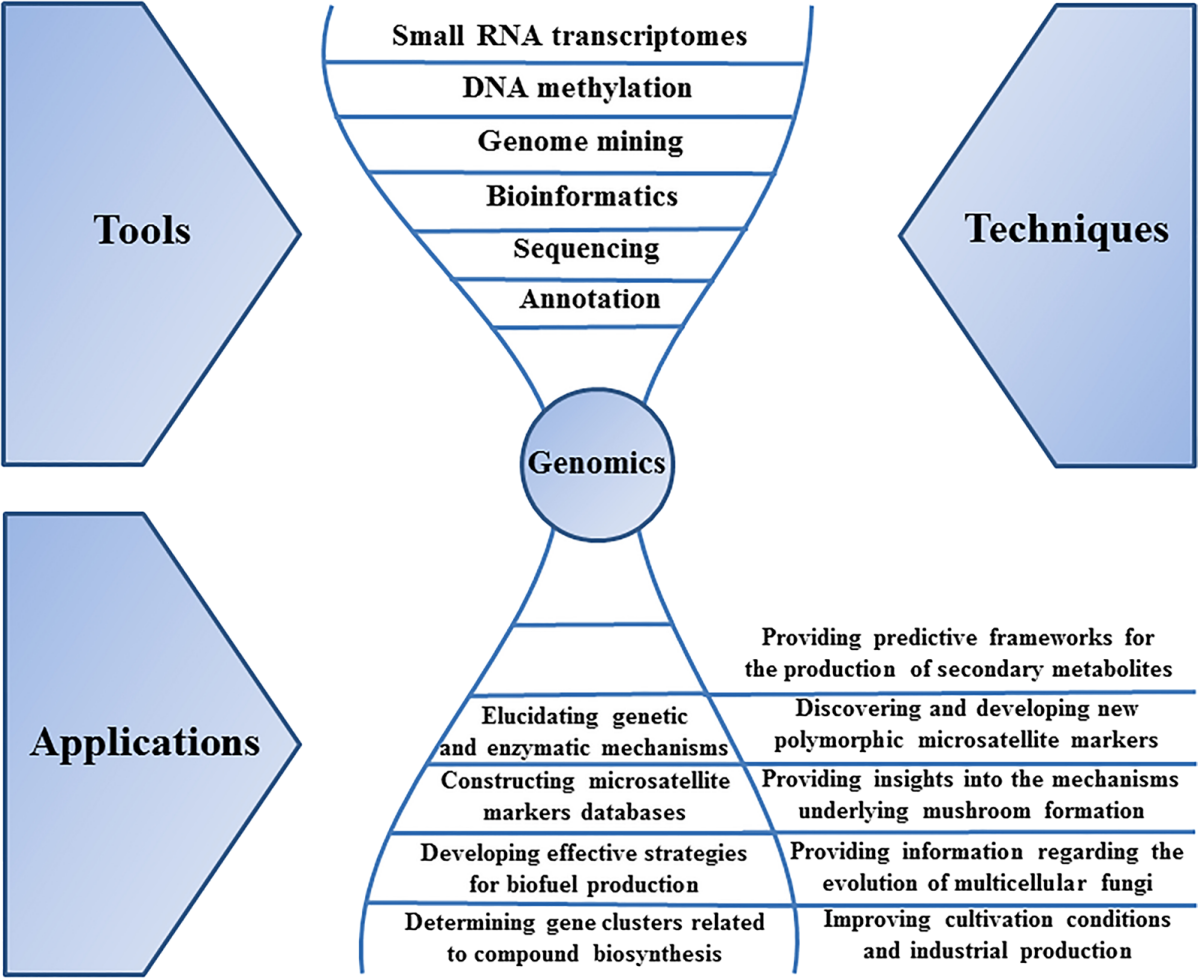
Commonly used techniques in genomics and a summary of the genomics applications in medicinal mushrooms

According to Table 2, genomics investigations have been an effective tool for studying medicinal mushrooms due to their roles in different subjects such as offering a genetic foundation of medicinal effects, improving biological and genetic studies, and elucidating genetic and enzymatic mechanisms in addition to biological characteristics related to different processes. Some of these processes are adaptation, degradation, sexual reproduction and development, sensitivity to different factors, mushroom formation, ethanol and medicinal compounds production, defense, evolutionary origins, and symbiosis. A summary of these applications as well as common techniques employed in genomics studies is demonstrated in Fig. 1. Also, information regarding the main techniques in genomics investigations, their different approaches, advantages, and limitations are provided in Table 3. For instance, FGENESH is the most rapid hidden Markov model-based program for precise ab initio gene structure prediction. When single-gene sequences are studied by this program, about 93% of all coding exon bases, along with 80% of human exons, can be predicted in 1.5 min. However, it is not as accurate as homology-based programs such as Exonerate and DIALIGN [149].

**Table 3:** Main techniques in genomics investigations, their different approaches, advantages, and limitations

Genomics techniques	Approaches and tools	Description	Advantages	Constraints	References
Genome mining	Southern blots	Is considered a classical approach Probes are created on the basis of conserved sequences and then these probes are used for screening for BGCs	Can be used for screening novel BGCs	Time-consuming	[150]
	In silico nucleotide/amino acid sequence alignment tools (e.g. BLAST, Diamond, and HMMer)	Mining for BGCs in databases (such as ClustScan and NP.searcher) and genome sequences is enabled by using a conserved sequence	Can be used for screening novel BGCs	Is mostly restricted to identifying particular classes of metabolites, including polyketide synthases and non-ribosomal peptide synthetases	
	In silico software (e.g. PRISM and antiSMASH)	Sequence alignment-based profiles in a Hidden Markov Model of genes specific for particular types of BGCs are exploited for anticipating BGC types	User-friendly	Only similar BGCs to previously recognized pathways can be identified	
	Machine learning- based mining tools	Machine learning strategies like ClusterFinder and DeepBGC are employed The set of known clusters or a set of clusters anticipated by in silico software are used for training these tools	Can be used for discovering unknown BGCs	The rate of false-positive results is much higher in comparison to in silico software Identifying completely novel BGCs is difficult	
					[150]
Genome sequencing	Shotgun sequencing	Sanger sequencing is used for gaining reads containing overlapping ends from smaller fragments of DNA. These overlapping ends will then guide the assembly of the obtained reads to achieve the original target sequence	Routinely utilized for short reads	Utilizes large amounts of data that are filled with sequencing errors Assembly of complex genomes is challenging Needs numerous overlapping reads for each fragment of the original DNA	[151,152]
	Whole-genome Shotgun sequencing	Genomic DNA is cut into random fragments, size-selected, and cloned into a suitable vector. The Sanger method is used for sequencing clones containing short inserts from both ends (paired-end sequencing). Sequence assembly software will then provide the original sequences from these reads	May be used for enhancing the accuracy of pre-existing sequencing information May be beneficial for revising or removing data gaps left by other DNA sequencing technologies	More prominent risk of long-range misassemblies and the emergence of sequencing errors Requires software	[153–155]
	Next generation sequencing (e.g. Roche 454, Illumina, etc.)	Delivers large amounts of high-throughput information from multiple samples per run in parallel	Fast Less expensive Delivers better coverage	Needs intricate bioinformatics interpretations Requires considerable data storage	[156]
Genome annotation	Ab initio	Depends on statistical models and gene signals Uses programs such as AUGUSTUS, FGENESH, etc	Identifies novel genes quickly and easily	Less accurate compared to Homology-based approaches	[149]
	Homology-based	Depends on sequence alignments Collects data from coding sequences, proteins, expressed sequence tags, and cDNA Uses programs and tools such as Exonerate, DIALIGN, etc	More accurate compared to Ab initio approaches Beneficial for functional annotations	Determining genes different from the referenced genes is difficult Increased evolutionary distance lowers its accuracy	

*BLAST* basic local alignment search tool, *PRISM* protein informatics system for modeling

The genomic studies, which are focused on determining biosynthetic pathways or biosynthetic gene clusters (BGCs) of bioactive metabolites and thus, can be considered more facilitative for increasing the production of these compounds, are discussed below in more detail.

### Genomics studies for exploring BGCs

Recent progress in genome sequencing indicates that many putative BGCs are not visible in fungal genomes [157,158]. However, platforms for advanced genome mining, which are beneficial for exploring BGCs of natural bioactive compounds generated from multi-enzyme pathways, can be provided by the existing mushroom genomes [159].

Clearly, genome mining is able to be employed for discovering the biosynthetic genes of formerly acknowledged products as well as new, unfamiliar products by different techniques such as whole-genome comparisons and genome search approaches [159]. A large number of unprecedented fungal metabolic gene clusters determined through genome mining initially seem to be silent (called cryptic or orphan BCGs) and incapable of producing desirable metabolic products. Still, some approaches have become available for the activation of these silent gene clusters via the utilization of different stress types and co-culturing with bacteria [160] or other fungi [161,162]. Subsequently, stimulated gene expression can be further investigated via transcriptomics, proteomics studies, metabolomics [160,162–170], and co-expression correlations [171,172]. In addition to genome mining efforts, advancements in bioinformatics software, including antiSMASH, PRISM, and SMURF, have made the understanding of suppression or activation of microbial biosynthetic pathways possible [173].

Chen et al*.* determined the *H. erinaceus* gene clusters that participated in bioactive secondary metabolites biosynthesis (e.g., terpenoid and polyketides biosynthesis) by conducting genomic analyses including multiple sequence alignments, phylogenetic investigations, and using software such as antiSMASH [10]. Indeed, the prediction of three gene clusters associated with terpene production and one gene cluster relating to polyketides biosynthesis (PKS) in *H. erinaceus* resulted in discovering a novel family of diterpene cyclases in this fungus [10,174]. These results can make uncovering and production of valuable secondary metabolites of *H. erinaceus* and other medicinal mushrooms easier in the future and offer useful data for secondary metabolite exploration in other basidiomycetes. *F. filiformis*, with the genome length of 35.01 Mb and 10,396 gene models, was predicted to have thirteen putative terpenoid gene clusters, 12 sesquiterpene synthase genes from four different categories, and two type I polyketide synthase gene clusters in its genome. In comparison to its cultivar strain (81 genes), more terpenoid biosynthesis-associated genes were existent in the wild strain (119 genes) [61]. Moreover, the wild strain of *F. filiformis* has more terpenoid and polyketide synthase gene clusters compared to *H. erinaceus*. In another study, a distinct network of sesquiterpene synthases and two metabolic gene clusters, which contribute to illudin sesquiterpenoids biosynthesis, were demonstrated by the draft genome sequence of *Omphalotus olearius.* As a holistic survey of all currently available Basidiomycota genomes became possible through characterizing the sesquiterpene synthases, a prognosticative resource for biosynthesizing terpenoid natural products was presented in these mushrooms [148]. These findings will be a great help in the discovery and biosynthesis of peculiar pharmacologically relevant substances from Basidiomycota.

### Genomics studies with the aim of elucidating biosynthetic pathways

Undoubtedly, studying the genome of medicinal mushrooms is effective for promoting research and development in pharmacological and industrial fields [129]. For instance, 16 cytochrome P450 superfamilies, possibly involved in the terpenoid synthesis, were detected by sequencing analysis of the *G. lucidum* genome via whole-genome shotgun strategy [129,136]. Detection of these superfamilies helped in determining the ganoderic acid synthetic pathway, massively producing triterpenoids, and achieving heterogonous expression through synthetic biotechnology. Moreover, a study on *G. lucidum* by Liu et al. indicated the genes associated with wood degradation and triterpene biosynthesis by comprehensive annotation of analyzed genes from the genome [137]. Regarding the model medicinal fungus, *G.sinense*, a comprehensive outline of its secondary metabolism and defense mechanisms was achieved through the investigation of DNA methylation patterns, small RNA transcriptomes, and complete genome sequence [139]. Thus, sequencing analysis, gene annotation, examining small RNA transcriptomes, and patterns of DNA methylation might be suitable techniques in concentrating genomic studies on secondary metabolite biosynthesis in the *Ganoderma* genus. Small RNA transcriptome analysis has not resulted in the overproduction of bioactive metabolites in *G.lucidum* yet. However, it was formerly demonstrated that microRNAs (miRNAs) could regulate secondary metabolite biosynthesis in many plants [175,176]. Hence, conducting small RNA profiling for determining miRNAs, studying miRNA-dependent regulation of valuable metabolites, and investigating miRNAs targeting genes associated with biosynthetic pathways can assist us in designing metabolic engineering strategies to improve bioactive substance contents in the desired organism.

As mentioned before (Sec “Background” section), increasing the production of a medicinal compound is not possible without having knowledge of its biosynthetic pathways and regulation. As the genes, pathways, and procedures related to the biosynthesis of the bioactive substances and wood decay by *S. sanghuang* were unidentified, Shao et al*.* investigated and reported a 34.5 Mb genome encoding 11,310 predicted genes of *S. sanghuang*. In this study, homologous genes associated with the biosynthesis of triterpenoids, polysaccharides, and flavonoids were determined. Then, the expression of these genes was investigated throughout four phases of development (10 and 20 days old mycelia, one-year-old fruiting bodies, and fruit bodies with three years of age). Furthermore, 343 transporters and four proteins of the velvet family, which were taking part in modulation, uptake, and redistribution of secondary metabolites, were detected [140]. As a result, genomics analysis can enhance our knowledge about secondary metabolites and their synthesis, which can be helpful for examining the medical applications of bioactive compounds and increasing their production in the future.

Not only the biosynthesis of sesquiterpenoids, antrocamphin, antroquinonol, ergostanes, and triterpenoids but also sexual development was clarified by exploiting genome ontology enrichment and pathway investigations in *A. cinnamomea.* Moreover, a 32.15-Mb draft genome including 9254 genes was achieved for this mushroom [97]. Also, the genome of *H. erinaceus*, which is consisted of 9895 genes, is 39.35 Mb and conveys different enzymes and a huge family of cytochrome P450 (CYP) proteins contributing to terpenoid backbones, sesquiterpenes, diterpenoids, and polyketides biosynthesis [10]. As another example, the obtained information from genome sequencing of *C. militaris* can significantly improve molecular research on the biology, fungal sex, and pathogenicity of this mushroom, uncover its mechanisms of medicinal compound synthesis, and be effective in the commercial production of its fruiting structures. In fact, utilizing the medicinal compounds of this mushroom can be facilitated by exploiting genome sequence data [138]. It is also worth mentioning that throughout the subculture and storage, *C. militaris* can experience a high frequency of strain degeneration which restricts the large-scale production of its bioactive compounds. In this case, genome-wide analysis of DNA methylation has shed light on the possible degeneration mechanisms of this strain [163] which will be beneficial for facilitating large-scale metabolite production. Regarding DNA methylation analysis, it is possible that the methylome repositories of *P. tuoliensis and P. eryngii* var. *eryngii* ease future investigations of epigenetic regulatory mechanisms supporting gene expression throughout the development of mushrooms. Thus, these repositories may have the potential to be considered as a guide for selecting the most suitable lifecycle/developmental phase for overproducing desired metabolites in medicinal mushrooms [164].

The genetic basis of the therapeutic activities of *L. rhinocerotis,* a comparative genomics source for polyporoid fungi and a platform for further identification of putative bioactive proteins and pathway enzymes of secondary metabolites is offered by the genome content of this mushroom [135]. By obtaining more information regarding biosynthetic pathways via genomic analyses, more targets for metabolic and pathway engineering can be found, which eventually contribute to rational predictions in the production of desired bioactive compounds.

Hitherto, more insights into the gene clusters or biosynthetic pathways of triterpenoids, ganoderic acids, polysaccharides, flavonoids, sesquiterpenoids, ergostanes, antroquinonol, antrocamphin, and polyketides in medicinal mushrooms have been achieved through genomic studies. Indeed, genomic investigations and genome sequencing programs are considered remarkable resource providers for determining new genes which contribute to the synthesis of bioactive substances (both known and novel substances). Also, more medicinal mushroom genomes will continue to become available [159]. Thus, progress in genome sequencing and genomic studies, genome mining, and bioinformatics, along with the availability of more genomes can greatly assist us in understanding the metabolic functions of desired organisms, which may result in both novel compound identification and improving the production of previously known valuable substances.

## Transcriptomics studies on different medicinal mushrooms

The set of all RNA molecules, including mRNA and non-coding RNAs, which are transcribed in one cell or a population of cells, is defined as the transcriptome. In other words, it is the complete transcript set in a specified organism or a particular transcript subset in a specific type of cell. Although genomes of a given cell line are not changeable, external environmental conditions may cause the transcriptome to alter considerably. Because transcriptome includes every cellular mRNA transcript, it reveals the genes actively expressed at any particular moment, excluding mRNA degradation events [129].

In fact, expression profiling, together with advanced next-generation sequencing technology referred to as RNA sequencing (RNA-Seq) technology [177] and bioinformatics infrastructure, is among the most promising procedures for determining responsive genes, their modes of regulation, and related transcription factors in adaptation to certain abiotic and biotic components during a change in metabolism. In other words, in order to perform transcriptomic analysis at the level of nucleotides, high-throughput methods on the basis of DNA microarray technology or RNA-Seq are often used [129]. RNA-Seq allows the easy detection of rare and low-abundance transcripts, single-nucleotide polymorphisms, rare mutations and previously unknown gene isoforms, microbial RNAs, and regulatory micro-RNAs while microarray technology makes the parallel quantification of thousands of genes from various samples possible [178,179]. In addition, using Illumina sequencing technology has paved the way for de novo transcriptome assembly and analyzing gene expression even in species with no full genome data [180]*.*

Transcriptomic analysis has been done in higher fungi [129], including different medicinal mushrooms such as *C. militaris* [181], *G.lucidum* [182]*, V.volvacea* [183], *P. ostreatus* [184], *Ophiocordyceps sinensis* (*Cordyceps sinensis*) [185], *H. erinaceus* [10]*, F. filiformis* [61]*, A. cinnamomea* [97], *P. eryngii* [186], *Termitomyces albuminosus* [187], *L. edodes* [188], and *L. rhinocerotis* [169]. For instance, genome-wide transcriptome analysis was conducted on different developmental stages of artificially cultivated *C. militaris* and uncovered 2712 differentially expressed genes between its mycelium and fruiting body [181]. Moreover, as the result of performing developmental transcriptomics on *O.sinensis*, key pathways and hub genes in the development of this mushroom as well as the gene profile related to its sexual development was better understood, which adds novel data to current models of fruiting body development in edible fungi [189]. Also, Zhu et al*.* discovered 8906 potential RNA-editing sites in *G. lucidum* at the genomic level and the genes consisting of RNA-editing sites were functionally categorized by the Kyoto encyclopedia of genes and genomes (KEGG) enrichment and gene ontology analysis. As a result, laccase genes contributing to lignin degradation, key enzymes involved in triterpenoid biosynthesis, and transcription factors were enriched. Furthermore, the influence of transcriptional plasticity on the mushroom development and growth as well as on the adjustment of secondary metabolic biosynthetic pathways was elucidated [190].

Therefore, transcriptome analyses can provide a better understanding of gene expression changes in different developmental stages in medicinal mushrooms. Also, various processes have been clarified through transcriptomics. For instance, regarding *P. ostreatus*, genome and transcriptome analysis gave insights into the decay process in postharvest mushrooms and indicated the application of high-throughput techniques for establishing models of living organisms exposed to different environmental conditions [184]. In another study, the functional genes of the terpenoid biosynthesis pathway and wood degradation in *G. lucidum* were demonstrated by analyzing transcriptome through Illumina high-throughput technology [180]*.* Hence, the obtained transcriptome datasets offer a platform of beneficial public information for future functional genomics studies relating to medicinal mushrooms [188] and can set the stage for choosing the most suitable lifecycle/developmental phase for achieving better and increased production of desired compounds. On the other hand, RNA-Seq along with systems biology tools (such as genome-scale metabolic networks) enables the systematic recognition of reporter metabolites that represent important regions of the metabolic network [191] and hot spots regarding metabolic regulation [192,193]. Thus, these tools can also be advantageous for discovering candidate targets for metabolic engineering purposes. Indeed, by adopting systems approaches, we can initiate experiments toward strain improvement to gain enhanced production of fungal metabolites. Also, this enhancement can be achieved via different routes ranging from maneuvering on cultivation medium to manipulating the cellular metabolic regulation. Some transcriptomic findings related to the biosynthesis of bioactive compounds and the development of their production are discussed below.

### Transcriptomics studies focused on cordycepin biosynthesis

The transcriptome of *O. sinensis* was investigated by Xiang et al. Examining adenosine kinase, 5′-nucleotidase, and adenylate kinase, which are possibly associated with the phosphorylation and dephosphorylation in the biosynthesis of cordycepin, offered valuable data for elucidating the cordycepin biosynthetic pathway. A model for cordycepin synthesis was also achieved [185]. This study offers a transcriptome dataset that can be considered a new resource for discovering genes (such as mating-type genes and genes associated with modulating signal transduction and the level of transcription in fruiting body development) besides examining and illuminating important biosynthetic and developmental pathways not only in *O. sinensis* but also in other medicinal mushrooms.

Although the metabolic pathways that contribute to the production of cordycepin were acknowledged to be linked to different carbon sources, the cellular regulatory procedures at the systems level were not well described [192]. Therefore, transcriptomic and genome-scale network-driven analyses were performed in *C. militaris* strain TBRC6039 cultivated on sucrose, glucose, and xylose carbon sources in order to examine the global metabolic response to the biosynthesis of cordycepin. Identification of 2883 DEGs, which were about 17% of the total 16,805 expressed genes, revealed sucrose and glucose-mediated alterations in the transcriptional regulation of central carbon metabolism (CCM). Also, reporter metabolites and main metabolic subnetworks including methionine, adenosine, and cordycepin, were offered via up-regulating cordycepin biosynthetic genes and after exploiting genome-scale metabolic network-driven analysis. These results present valuable data regarding *C. militaris* for systems-wide cordycepin overproduction [192] and indicate that the applied techniques, transcriptomics combined with genome-scale network-driven investigations, should also be extended to other higher fungi and other bioactive compounds in order to facilitate overproduction. Since *C. militaris* genome and RNA-sequencing data are available, integrating data for the investigation of cellular metabolism underlying cordycepin production has become possible [194]. Thus, the responsive mechanism of xylose consumption in *C. militaris* strain TBRC7358, the precursor and energy resources for cell growth and cordycepin production, and a remarkable role of putative alternative pathways for providing cordycepin production precursors on xylose were indicated by DEGs and the reporter metabolites analysis [195]. Enhancement of the cultivation procedure for increasing cordycepin and biomass productivities can be done with the help of the insight gained from this study which sheds light on the molecular mechanism underlying main metabolic pathways in transferring xylose towards cordycepin biosynthesis in *C. militaris* TBRC7358 [195]. These outcomes indicate that employing transcriptomic studies can clarify both main and alternative metabolic pathways related to the production of medicinal substances. Moreover, based on previous studies, genes related to cordycepin biosynthesis were up-regulated by growing *C. militaris* in favorable carbon sources. So, cultivating *C. militaris* strains for growth and cordycepin production relied on favored carbon sources proposing the essentiality of systems design of cultivation medium [196,197].

Another transcriptome analysis was performed on a *C. militaris* with a two-fold enhancement of cordycepin production caused by adding l-alanine to gain a deeper insight into molecular procedures of l-alanine’s effect on cordycepin biosynthesis. This investigation resulted in the achievement of a metabolic network map from the substrate amino acid to the product cordycepin and it was demonstrated that the Zn2Cys6-type transcription factors contributed to the development of *C. militaris* fruiting [13] as well as the regulation of its secondary metabolites [198]. This study indicates the plasticity of the cordycepin network, identifies the genes of rate-limiting enzymes in energy production pathways and amino acid conversion, and provides a suitable basis for future improvement of strain breeding and cordycepin yield. Also, these methods can be used for determining the influence of other inducers on metabolite biosynthesis from the molecular point of view.

So far, different tools such as genome-scale metabolic models (GSMMs) and genome-scale network-driven analyses, computer-assisted tools, reporter metabolites analysis, and information gained from other omics investigations have proved to be prominent for transcriptomics studies in cordycepin-producing mushrooms. Combining these tools and integrating their resultant data may generate new strategies for overproducing cordycepin.

### Transcriptomics studies focused on the biosynthesis of other valuable bioactive compounds

In order to elucidate the biosynthetic pathway of carotenoids and its related genes, the transcriptomes of *C. militaris* mycelia grown under dark (CM10_D) and light exposure (CM10_L) conditions were sequenced and compared with each other. Furthermore, according to the KEGG pathway enrichment analysis of DEGs, most DEGs were elevated in “metabolic routes,” “MAPK signaling pathway-yeast,” and “secondary metabolite biosynthesis.” Also, the significant effect of the *Cmtns* gene in the biosynthesis of carotenoids was demonstrated in this mushroom [199]. Moreover, Yang et al. performed de novo sequencing and transcriptome investigation in the termite mushroom *T. albuminosus*, and their work resulted in the identification of enzymes related to saponin biosynthesis, including 22 glycosyltransferase and six cytochrome P450s genes [187]. As another example, the first transcriptome re-sequencing examination of *L. rhinocerotis* was performed by Yap et al*.*, which uncovered the expression of several secondary metabolite biosynthetic routes (especially biosynthesis of terpene) along with putative genes associated with the biosynthesis of sclerotium glucans. Genes that encoded the sugar-binding lectins, cysteine-rich cerato-platanins, and hydrophobins were some of the genes with the highest expression in the sclerotium [169].

### Role of comparative transcriptomics in medicinal compound overproduction

Profiling differences in gene expression covering different tissues of *H. erinaceus* (the monokaryotic mycelium (MK), dikaryotic mycelium (DK), and fruiting body) demonstrated the up-regulation of terpenoid biosynthesis-related genes in mycelia while the gene contributing to polyketides biosynthesis, experienced up-regulation in the fruiting body [10]. A similar study in *F. filiformis* revealed that contrary to *H. erinaceus*, a good number of terpenoid biosynthesis genes were up-regulated in the primordium and fruiting body of the wild strain, whereas polyketide synthase genes showed up-regulation in its mycelium. Relatively high transcript levels of UDP-glucose pyrophosphorylase and UDP-glucose dehydrogenase encoding genes, which are associated with the biosynthesis of polysaccharides, were observed in the mycelia as well as fruiting bodies [61]. In another study, DEGs between mycelia and fruiting bodies as well as 242 proteins in the mevalonate pathway, terpenoid pathways, polyketide synthases, and cytochrome P450s which may be related to the biosynthesis of secondary metabolites with therapeutic properties, were identified in *A. cinnamomea*. Expression enrichment was observed in genes of secondary metabolite routes for tissue-specific substances, such as 14-α-demethylase (CYP51F1) in the fruiting body for transforming lanostane to ergostane triterpenoids, coenzymes Q (COQ) for biosynthesizing antroquinonol in mycelium, and polyketide synthase for antrocamphin production in the fruiting body [97]. Tang et al. exploited RNA-seq technology for analyzing the poly (A) + transcriptome. They generated profiles for comparing the expression of Brown film (BF) and non-Brown film mycelia in order to elucidate the molecular mechanisms in *L. edodes* during light-induced BF formation. Through de novo assembly, a total of 31,511 contigs was achieved. Moreover, comparative analysis of the expression profiles demonstrated that prospective genes contributing to light-induced BF generation play important parts in fungal photoreception, the production of secondary metabolites, and signal transduction [188]. Henceforth, these findings can offer useful information for molecular breeding, selecting the best tissues/developmental stages with higher potential for producing elevated levels of the desired medicinal compounds, enhancing compound biosynthesis, and improvements in novel compound production through heterologous pathways and metabolic engineering. In addition, they will be advantageous for providing more insights into the mechanisms of gene expression and gene regulation besides further functional and pathway analysis.

In addition to determining DEGs among different tissues and developmental stages in an individual organism, comparative transcriptomics can be used for elucidating processes and gene expression differences among different culture conditions. For instance, *G. lucidum* goes through differentiation and morphological alterations in liquid static culture. This process, which results in the formation of aerial mycelia and asexual spores with substantial amounts of ganoderic acids, should be studied in order to allow large-scale production of asexual spores and ganoderic acids. Thus, comparative transcriptome analysis via suppression subtractive hybridization (SSH) method incorporated with cDNA array dot blotting was performed for identification of DEGs in liquid static culture contrasted with shaking culture of *G. lucidum*. Subsequently, 147 unigenes (such as unigenes regarding asexual sporulation and signal transduction) were detected in liquid static culture. Among these 147 unique sequences, protein database matches were identified for 101 (68.7%) expressed sequence tags (ESTs), 88 (59.8% of total) ESTs had considerable similarity to acknowledged proteins, and 13 (8.9% of total) sequences were comparable to hypothetical proteins. However, as there were slight resemblances to the recognized sequences for the remaining 46 ESTs (31.3%), they may demonstrate novel genes [200].

Based on the reviewed transcriptomics studies, it is exemplified that transcriptomic analyses are powerful tools that can be employed for several purposes, including enhancement of understanding about the functions and evolution of fungal genomes and the clarification of the molecular mechanisms of various cellular processes (e.g., mechanisms of gene expression and gene regulation). Furthermore, detection of reporter metabolites, investigation of the transcriptional response of desired organisms in the presence of different factors, and the determination of responsive genes, their modes of regulation, and related transcription factors can be facilitated by exploiting transcriptomic techniques.

**Fig. 2:**
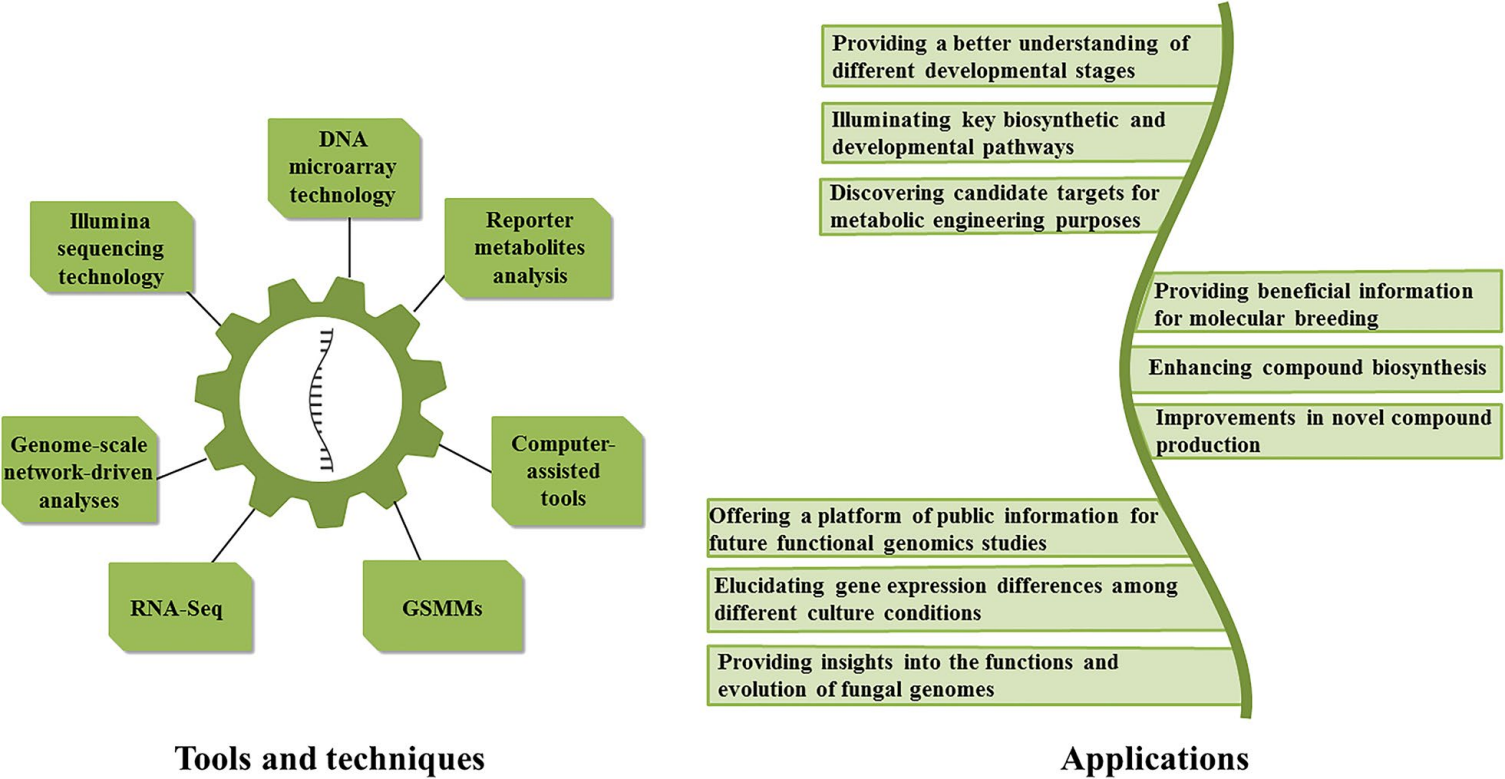
Commonly used techniques in transcriptomics and a summary of the transcriptomic applications in medicinal mushrooms

Other applications of these techniques include the discovery of the differences in gene expression between various developmental stages and different culture conditions, understanding the changes during the development, and determination of the functional genes, enzymes, and biosynthetic pathways associated with bioactive compounds production. Thus, data obtained from transcriptome studies will be beneficial for investigating functional genomics in medicinal mushrooms, molecular breeding, bioactive compounds overproduction, and improving the synthesis of novel substances via heterologous pathways and metabolic engineering. Common techniques used in transcriptomics studies and a summary of the applications of transcriptome analyses in medicinal mushrooms are provided in Fig. 2.

## Proteomics studies on different medicinal mushrooms

Methodical discovery and quantification of the complete protein set in a biologic system, namely cell, tissue, or organism, performed at a particular moment, are defined as proteomics analysis [129]. Proteome investigations can bring about a myriad of advantages. For instance, these studies are believed to be a suitable strategy for investigating mushroom developmental processes and understanding the roles of enzymes and proteins in prospective cultivation procedures, particularly in mushrooms with challenging cultivation conditions [201]. In addition to being helpful in better understanding the cellular metabolism [129], proteomics supports the identification of the reservoir of minerals and vitamins as well as protein effectors in mushrooms which possibly possess antibiotic, antitumor, antioxidant, antidiabetic, apoptosis, and blood pressure management effects [201]. Also, it is an effective tool for determining quantitative alterations in protein expression of filamentous fungi in reaction to stress exposure [202]. However, identifying all protein spots is not possible via proteomic analysis [129]. Thus, they should be exploited along with other omics studies. Proteomics techniques, including 2-dimensional gel electrophoresis (2-DE) or liquid chromatography coupled with mass spectrometry (LC − MS) (known as standard proteomic approaches) [203], 2DE gel-based proteomics [201], difference gel electrophoresis (DIGE) technology [204], LC-based techniques particularly high-throughput shotgun proteomics [205], gel-free proteomics [206], and iTRAQ labeling technique incorporated with two-dimensional liquid chromatography-tandem mass spectrometry (2D LC − MS/MS) [202], have turned into essential complements to genome and transcriptome techniques in fungal biology [207]. Moreover, 2DE gel-based proteomics is considered the most effective and commonly used technique for investigating fundamental physiological subjects in fungi, especially in edible mushrooms [201].

Proteomic analysis has been performed in different mushrooms such as *L*. *rhinocerotis* [208], *T. heimii* [209], *A. bisporus* [210], *Pleurotus tuber*-*regium* [211], *A. cinnamomea* [212], *G. lucidum* [170], *P. ostreatus* [213], and *F. velutipes* [214]. For instance, proteomic investigation of antihypertensive proteins was conducted in some edible mushrooms such as *A. bisporus* [210]*.* From another perspective, by examining protein expression profiles in different growth and developmental stages, a basis for the evaluation and comparison of these stages is offered in higher fungi. For example, information about biological processes contributing to the development of *T. heimii* w*as* provided by exploiting the proteomic method of 2D-DIGE for the identification and investigation of the protein profiles of each developmental stage [209]. Moreover, protein fractions of three developmental stages in *G.lucidum* were analyzed by LC–MS/MS, and expression of a possibly novel highly immunomodulatory protein was indicated [170]. These comparative studies have also been conducted on *P. tuber*-*regium* [211] and *A. cinnamomea* [212].

Hence, both developmental stage assessment and novel mushroom compound identification can be achieved using proteomic techniques. Furthermore, analyzing changes in protein expression between two different mushroom species can be viewed as another application of proteomics that results in uncovering unique properties of individual organisms and eventually will be helpful in the detection of key compounds in their metabolisms. However, proteomic analysis is still in the early and developmental stages in higher fungi and edible mushrooms in comparison to bacterial, plant, and human proteomics investigations as a result of experiment costs and whether complete genome sequences of the mushrooms are available or not [129,201]. Nevertheless, proteomic studies have been executed on these organisms, including *Pleurotus* species, *G. lucidum*, and *F. velutipes*, aiming to improve bioactive metabolite production. These studies will be described below.

### Proteomics studies in *Pleurotus* species

Apparently, the *Pleurotus* species is considered the most investigated genus of edible mushrooms in the proteomic subject area since it is among the most extensively cultured edible mushrooms [201]. Mycelial growth is limited in the presence of lignin in agro-industrial residues because of the intricate structure of the substrate and complications in using polysaccharides. Thus, investigating lignocellulose-fungi interactions is prominent for becoming aware of the ecology of fungi and optimizing the bioconversion of agro-industrial substrates to biotechnologically important products [34]. Attempts have been made in order to examine the procedure of the lignocellulose-fungi interactions via proteomic studies. For instance, the proteomic profile of *P. ostreatus* cultivated with different lignocellulose substrates as well as differentially expressed intracellular proteins in these substrates were reported by Xiao et al*.*, which helped in studying the metabolic pathways associated with lignocellulose response in *P. ostreatus*. Also, 115 proteins were detected and it was demonstrated that enzymes contributing to sugar transformation via different metabolic routes experienced enhancement, and better growth was observed in the presence of xylan and carboxymethylcellulose [213]. In addition to *P. ostreatus*, these findings can also be useful for other white-rot fungi.

It was previously observed that applying Tween 80 to a submerged fermentation procedure can improve mycelial growth and the production of exopolysaccharides in *P. tuber-regium* by 51 and 42%, respectively [215]. Thus, a proteomic analysis was performed on this mushroom in order to identify the influence of stimulating agents (Tween 80) on mycelial growth and the production of exopolysaccharides in liquid culture. According to the results, a positive regulation on heat shock proteins (assist in maintaining cell viability under stressful circumstances) as well as on two isoforms of ATP-citrate lyase (can impede the Tricarboxylic acid (TCA) cycle activity and thereby increase exopolysaccharide biosynthesis) was detected. In fact, 32 proteins, which were expressed differentially, were determined by one-dimensional gel electrophoresis, and ATP: citrate lyase isoform 2 was able to increase exopolysaccharide production [216]. In addition to filling the information gap in the underdeveloped field of mushroom proteomics, these findings can explain how stimulatory agents, such as Tween 80, can improve the biosynthesis of beneficial compounds.

### Proteomics studies in *G. lucidum*

Under nitrogen-limiting fermentation conditions, metabolic rearrangements take place due to the induction of growth inhibition via autophagy and imbalances between carbon (C) and N. These rearrangements adjust the division of cells, morphology, and lipids and starch cumulation processes in order to keep cellular structures safe and raise the survival probability. Since nitrogen (N) limitation is a suitable method for increasing ganoderic triterpenoid (GT) accumulation in *G. lucidum*, Lian et al. analyzed the dynamic adjustment of metabolism reallocation towards GT production in response to N limitation through exploiting iTRAQ-based proteome. Also, they attempted to identify the fundamental molecular mechanisms of the positive effect of N-limiting conditions on achieving high GT concentrations. As a result of applying N-limiting conditions, several changes were observed; (1) cell division ceased possibly due to the occurrence of autophagy, and cells modified their physiological and metabolic activities to compensate for the nutrient limitation; (2) N limitation did not affect cell growth tremendously but caused a considerable increase in GT amounts in the first 20 days. From the 10th day, extended duration of N limitation halted protein contents; (3) biosynthesis of nitrogen-containing substances experienced a decrease; (4) the generation of acetyl-CoA was promoted via metabolic reprogramming of CCM, which may supply GT biosynthesis; (5) in addition to up-regulation of enzymes involved in protein degradation, protein regulation in response to the abiotic stress and oxidation–reduction procedures carried out an important role in retaining cellular homeostasis; (6) while ongoing N limitation raised the mycelial contents of GT, it lowered biomass production of *G. lucidum*.

The obtained results show that the flux of carbon to GT in N deficient conditions resulted from the intermediary metabolism remodeling in the TCA cycle and glycolysis reactions. *G. lucidum* may utilize mechanisms such as glycolysis reinforcement and diminishment of other pathways in CCM to increase carbon flux solely toward secondary metabolites. Proteomics-based analyses, which helped in constructing a network of metabolism reallocation toward GT, demonstrated that glycolysis and the TCA cycle produce the carbon skeletons consolidated into GT precursors. Also, a basis for genetic engineering is offered by this study, which can allow the simultaneous synthesis of biomass and GT in *G. lucidum* [217]. These results may pave the way for establishing networks of metabolism reassignment toward bioactive compounds in other medicinal mushrooms, as well.

### Proteomics studies in *F. velutipes*

Liu et al*.* applied iTRAQ labeling combined with the 2D LC − MS/MS method for determining the overall chronological alterations in patterns of protein expression and the mechanism of regulation of *F. velutipes* mycelia in reaction to light and cold stresses. Among the 1046 nonredundant identified proteins, 264 distinctively expressed proteins were related to 176 certain KEGG pathways. Based on comprehensive data analysis, the regulatory network underlying the mycelial light and cold reaction processes of *F. velutipes* was complicated and multi-dimensional. The reason behind this complexity was that it included different activities like quick energy supply, production of different compounds (lysine, γ-aminobutyric acid, phenylalanine, tyrosine), and calcium signal transduction procedure. Moreover, generating dynein-dependent actin and microtubule cytoskeleton, self-digestion, morphogenesis of organs and tissues, pigment secretion, acclimatization to oxidative stress, and other processes related to stress contribute to this complication [214]. In addition to being helpful for scientifically improving some mushroom cultivation techniques, this information may lead to a deeper understanding of the stress response mechanisms in macro-fungi.

**Fig. 3:**
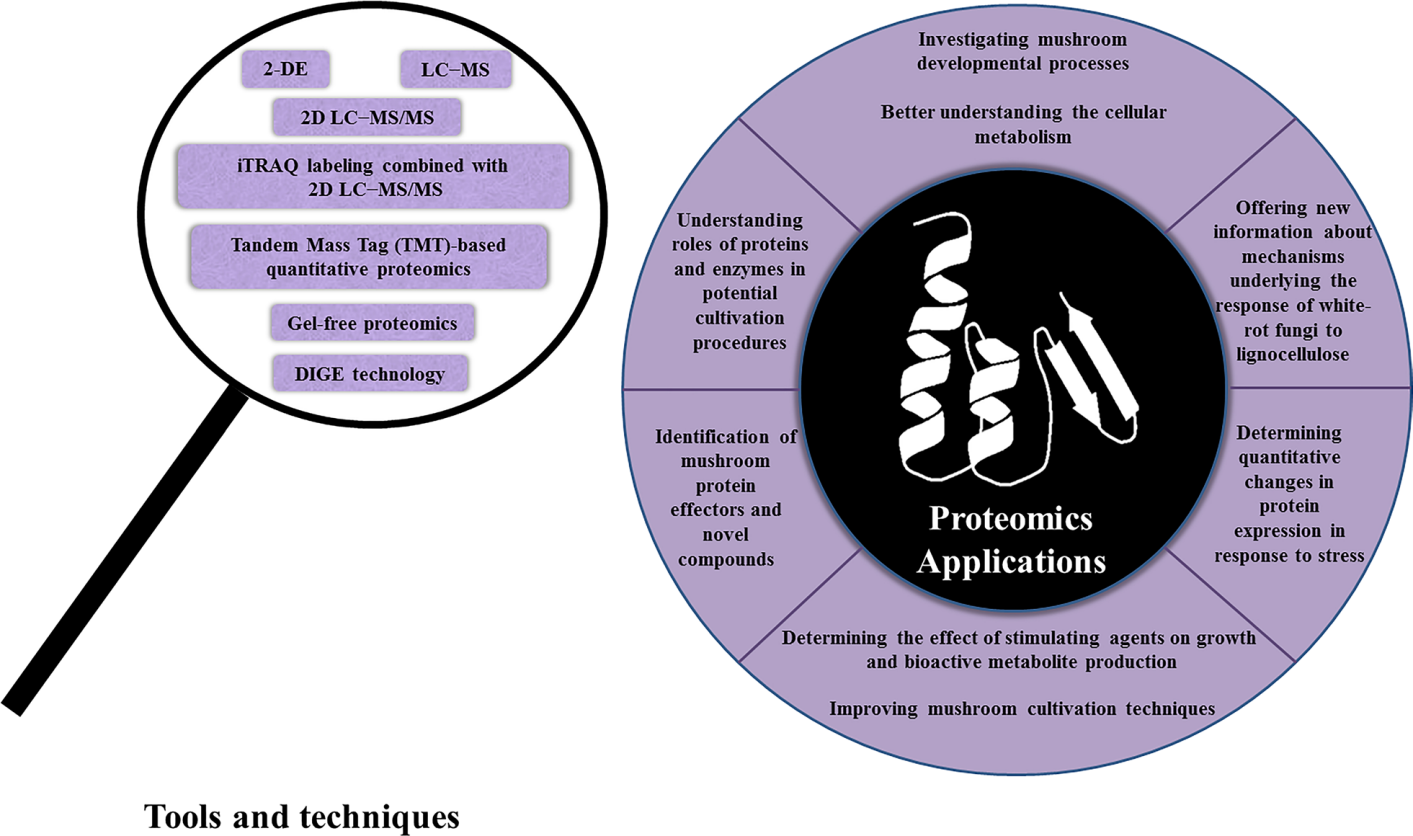
Commonly used techniques in proteomics and a summary of the proteomics applications in medicinal mushrooms

According to the studies mentioned above, proteomics investigations can be utilized for different aims such as analyzing the developmental processes of mushrooms and their associated candidate genes and signaling pathways, examining fundamental physiological subjects, and establishing networks of metabolism reassignment toward bioactive metabolites. Other implementations of proteomic analyses are depicted in Fig. 3. Also, detecting quantitative changes in protein expression of filamentous fungi in response to stress or different factors, explaining the mechanism underlying these responses and their associated metabolic pathways is possible by employing these investigations. Thus, proteomics has become a necessary complement to genome and transcriptome techniques.

## Combining transcriptomics and proteomics

Gene and protein expression profiling of medicinal mushrooms have helped in gaining knowledge about the genes and proteins involved in exogenous nutrient bag decomposition in *Morchella importuna* [218], temperature stress in *L. edodes* [219], bioactive metabolites in *H. erinaceus* [220], Cd^2+^ stress in *P. eryngii* [186], generation of special odor in *S. commune* [221], and development of the fruiting body in *F. velutipes* [222] and *D. indusiata* [223]. For instance, the study on *P. eryngii* indicated the coincidence of secondary metabolite production inhibition with the increase in carbohydrate metabolism and the rate of energy [186]. Transcriptomic and proteomic studies were also performed on a dikaryotic strain (DK13 × 3) that were emerged from two monokaryotic *P. ostreatus* strains (MK13 and MK3)*.* This study offered evidence that growing a dikaryon organism is more advantageous than a monokaryon because the genes contributing to the utilization of macromolecules, cellular material synthesis, ability to withstand stress, and signal transduction had more regulation in the dikaryotic strain compared to MK13 and MK3 strains [224]. Thus, it will be possible to improve the characteristics of the strains and make them more resistant to the environment by selecting monokaryon organisms and doing the crosslink. As a result, the desired improvements will be observable in the formation of the dikaryon. The transcriptomic examinations and transcriptomics combined with proteomic studies on medicinal mushrooms are summarized in Table 4.

**Table 4:** Summary of transcriptomics studies/transcriptomics combined with proteomics studies on medicinal mushrooms

Mushroom	Omics study	The investigated processes/fields	Results/ Proposed applications	References
*C. militaris*	Genome-wide transcriptome and proteome investigation	Variations in gene expression between the mycelia and fruiting bodies	Gene expression comparison in different developmental stages	[181]
*C. militaris*	Transcriptomics	•L-alanine’s effect on cordycepin biosynthesis at a molecular level •Genes of rate-limiting enzymes in energy production pathways and amino acid conversion •Cordycepin network	Uncovering mechanisms, their associated genes, and transcription factors Achieving metabolic network maps from the substrate to the desired product Increasing the yields of bioactive metabolites and improving strain breeding	[198]
*C. militaris*	Transcriptomics	•Carotenoids production •Biosynthetic pathway of carotenoids and its related genes	Improving the production of bioactive metabolites Elucidating biosynthetic pathways of bioactive metabolites and their associated genes	[199]
*Cordyceps kyushuensis*	Transcriptomics and proteomics	Gene clusters associated with the production of cordycepin and pentostatin	Identification of gene clusters and functional proteins associated with the production of bioactive metabolites Improving the yield of bioactive metabolites	[225]
*G. lucidum*	Comparative transcriptome analysis	•Comparing DEGs in liquid static culture and shaking culture •Synthesis of ganoderic acids and asexual spores	Providing beneficial data for large-scale synthesis of bioactive compounds	[200]
*G. lucidum*	Transcriptomics	•Mushroom development and growth •Adjustment of secondary metabolic biosynthetic pathways	Elucidating the effect of transcriptional plasticity	[190]
*G. lucidum*	Transcriptomics	Functional genes of the wood degradation and terpenoid pathway	Providing a basis for conducting functional genomics research	[180]
*G. lucidum*	Transcriptomics	Mitochondrial DNA	Offering more information about the functions and evolution of fungal mitochondrial DNA	[182]
Copper-induced lignocellulolytic *G.lucidum* MDU-7	Transcriptomics and proteomics	Lignocellulose breakdown and terpenoid biosynthetic routes	Uncovering genes related to different pathways	[226]
*V. volvacea*	Transcriptomics	Stipe development and switching from egg to elongation stage	Providing a better understanding of the changes during different developmental stages	[183]
*P. eryngii*	Transcriptomics	•Transcriptional response to considerable levels of heavy metals •Mechanism of NO in increasing heavy metal tolerance •Coincidence of secondary metabolite production inhibition with the increase in carbohydrate metabolism and the rate of energy	Offering data regarding transcriptional responses to different environmental conditions Uncovering mechanisms	[186]
*P. eryngii*	Transcriptomics and proteomics	Cd^2+^ stress	Providing insights into the genes and proteins related to different stressful conditions	[186]
*O.sinensis*	Developmental transcriptomics	Hub genes and main routes in the development process	Providing insights into the gene profiles related to sexual development	[189]
*O.sinensis*	Transcriptomics	Modulating signal transduction and the level of transcription in fruiting body development Cordycepin biosynthetic pathway	Providing models for the synthesis of bioactive metabolites Uncovering genes and investigating important biosynthetic and developmental pathways	[185]
*H. erinaceus*	Transcriptomics	Terpenoid biosynthesis in mycelia Polyketides biosynthesis in the fruiting body	Providing insights into the expression and regulation of biosynthetic genes in different developmental phases	[10]
*H. erinaceus*	Transcriptomics and proteomics	Regulation of bioactive metabolites	Offering information about the genes and proteins involved in the regulation of bioactive metabolites	[220]
*A. cinnamomea*	Transcriptomics	Production of secondary metabolites with medicinal properties	Identification of DEGs between fruiting bodies and mycelia Providing beneficial data for enhancing the production of valuable metabolites	[97]
*T. albuminosus*	Transcriptomics	Saponin biosynthesis	Identification of enzymes related to the biosynthesis of bioactive compounds	[187]
*L. edodes*	Transcriptomics	•Light-induced formation of Brown film (BF) •Secondary metabolite biosynthesis •Gene expression and gene regulation mechanisms	Uncovering molecular mechanisms Helping further functional and pathway analysis	[188]
*L. edodes*	Transcriptomics and proteomics	Temperature stress	Offering information about the genes and proteins associated with different stresses	[219]
*M. importuna*	Transcriptomics and proteomics	Decomposition of exogenous nutrient bag	Providing information about the genes and proteins	[218]
*S. commune*	Transcriptomics and proteomics	Special odor formation	Providing data regarding the genes and proteins	[221]
*F. velutipes*	Transcriptomics and proteomics	Development of the fruiting body	Providing information about the genes and proteins	[222]
*D. indusiata*	Transcriptomics and proteomics	Development of the fruiting body	Providing information about the genes and proteins	[223]
*T. guangdongense*	Transcriptomics and proteomics	•Development and growth •Regulation of terpenoid, polysaccharide, ergosterol, and adenosine production-related proteins during the development	Indicating the significance of post-transcriptional procedures Investigating mechanisms of development in fungi at the molecular level Improving cultivation techniques	[104]
*L. rhinocerotis*	Transcriptomics	Secondary metabolite routes and little cysteine-rich proteins in the sclerotium	Discovering genes with considerable expression	[169]
*Agrocybe aegerita*	Transcriptomics and proteomics	Production and synthetic pathways of polysaccharides and sterol	Elucidating biosynthetic pathways Providing beneficial data for constructing mushroom cell factories in the future	[129]

On the other hand, transcriptomics and proteomics investigations can pave the way for more developmental and medicinal research in mushrooms. For instance, a better understanding of changes during the morphological development of *D. indusiata* was achieved through de novo transcriptome assembly and shotgun proteomics of its fruiting bodies which resulted in the detection of 4380 proteins. Moreover, annotation and functional analysis of the determined proteins depicted their considerable increase in different activities such as small molecule synthetic and metabolic procedures [223].

High-throughput sequencing analysis was used to achieve transcriptomic and proteomic data with respect to mycelia and fruiting bodies of *Agrocybe aegerit*a. The results of this work, which were helpful in illuminating the polysaccharide and sterol biosynthetic pathways, denoted that the polysaccharide was produced in great amounts in the fruiting bodies [129]. This data can be applied for constructing mushroom cell factories in the future. As another example, even though the genome sequence of *T. guangdongense* was available, there was not enough information regarding the regulatory networks of its metabolite production routes and sporocarp development. Thus, Wang et al. analyzed the transcriptome and proteome at distinctive developmental phases of *T. guangdongense* and found 9076 expressed genes as well as 2040 proteins. Also, hub genes were identified by exploiting weighted gene co-expression network analysis (WGCNA). As there was a small correlation between the transcriptomics and proteomics information, post-transcriptional procedures seem important in the development and growth of this mushroom [104]. Also, the down-regulation of terpenoid, polysaccharide, ergosterol, and adenosine production-related proteins was demonstrated during its development.

With respect to *G.lucidum*, combining De novo transcriptome assembly and proteomic studies under copper stress conditions pointed out genes related to terpenoid production routes and the breakdown of lignocellulose. As a result, it was shown that inducible lignin oxidative enzymes and proteins associated with secondary metabolic routes are highly abundant. Furthermore, through increasing Cu^2+^ concentrations, lignocellulase secretion in addition to antioxidants production was enhanced and about a fourfold increase was observed in phenolics production [226].

Omics technologies have been effectively utilized for investigating molecular mechanisms in *Cordyceps* fungi. Transcriptomic and proteomic analyses in artificially cultivated *C. militaris* have demonstrated the variations in gene expression between its mycelia and sporocarps. 2113 genes showed up-regulation in mycelia while 599 up-regulated genes were identified in sporocarps. Therefore, as it was inferred that the cordycepin metabolism pathway may have a higher activity in the mycelium of *C. militaris*, it is favorable to use the mycelium of this mushroom for the large-scale production of cordycepin [181].

Moreover, the efficiency of cordycepin can be decreased as the result of in vivo conversion to 3′-deoxyinosine by adenosine deaminase. Since pentostatin is able to impede adenosine deaminase, blending pentostatin with cordycepin can improve this efficiency. Thus, by exploiting transcriptomic and proteomic analyses, Zhao et al*.* investigated and reported a single gene cluster (consisting of four genes) associated with the production of cordycepin and pentostatin in *Cordyceps kyushuensis*. This cluster is able to be used for enhancing cordycepin yield and identifying more functional proteins [225]. As these results may also be observable in other *Cordyceps* fungi, conducting similar investigations on this genus is worth considering for increasing cordycepin production.

Thus, in addition to being an asset to better understanding mushroom development and obtaining strains with improved properties or more resistance to the environment, novel aspects and more data in other areas can be revealed by applying combined omics investigations on macro-fungi, compared to single omics studies. Some of these areas are biosynthetic pathways of bioactive metabolites, changes in the level of amino acids and other nutrients/metabolites, roles of regulatory factors, regulation of expression and cellular processes along with their molecular mechanisms, and the importance of post-transcriptional processes. Therefore, these investigations can eventually be used for increasing the yield of bioactive substances.

## Metabolomics studies on different medicinal mushrooms

Since metabolome is dynamic and can be changed every second (similar to transcriptome and proteome), metabolic profiles are able to provide instant photos of the cell's physiological conditions [129]. Indeed, metabolomics is a high-throughput and novel approach [227] that can be applied to higher fungi in order to analyze, both qualitatively and quantitatively, their metabolome existent during a specific period or following induction in a specific condition. Moreover, this approach helps in understanding biological processes [227], determining variation in extrinsic and intrinsic environment perturbation response as well as various phenotypes by exploiting nuclear magnetic resonance (NMR) or combining mass spectrometry (MS) with other chemical analysis systems such as gas chromatography (GC/MS), HPLC (HPLC–MS), and capillary electrophoresis [228]. Metabolomics studies have been executed on *Cordyceps bassiana*, *Phanerochaete chrysosporium, T*.*versicolor*, *Dichomitus squalens*, *P*.*ostreatus*, and *D.indusiata.* Moreover, metabolite profiles have been exploited for chemotaxonomy [229] and for investigating different developmental phases or growth conditions in higher fungi. For instance, metabolic profiles of mycelia and fruiting bodies of *C. bassiana* were achieved via multivariate data analysis and H-1 NMR spectroscopy [230]. Also, measuring dynamic multi-parametric metabolic reactions of biological systems to genetic alterations or pathophysiological stimulants in a quantitative way is known as metabonomics. In fact, metabonomics is considered a subset of metabolomics [231] and is described as scientifically analyzing chemical processes including metabolites [129]. However, in order to arrive at more comprehensive conclusions, metabolomics study is regularly combined with other omics technologies such as proteomics and transcriptomics investigations [129]. For instance, metabolomic studies and proteomic investigations of the benzoic acid metabolism were carried out in *P. chrysosporium* [232].

**Fig. 4:**
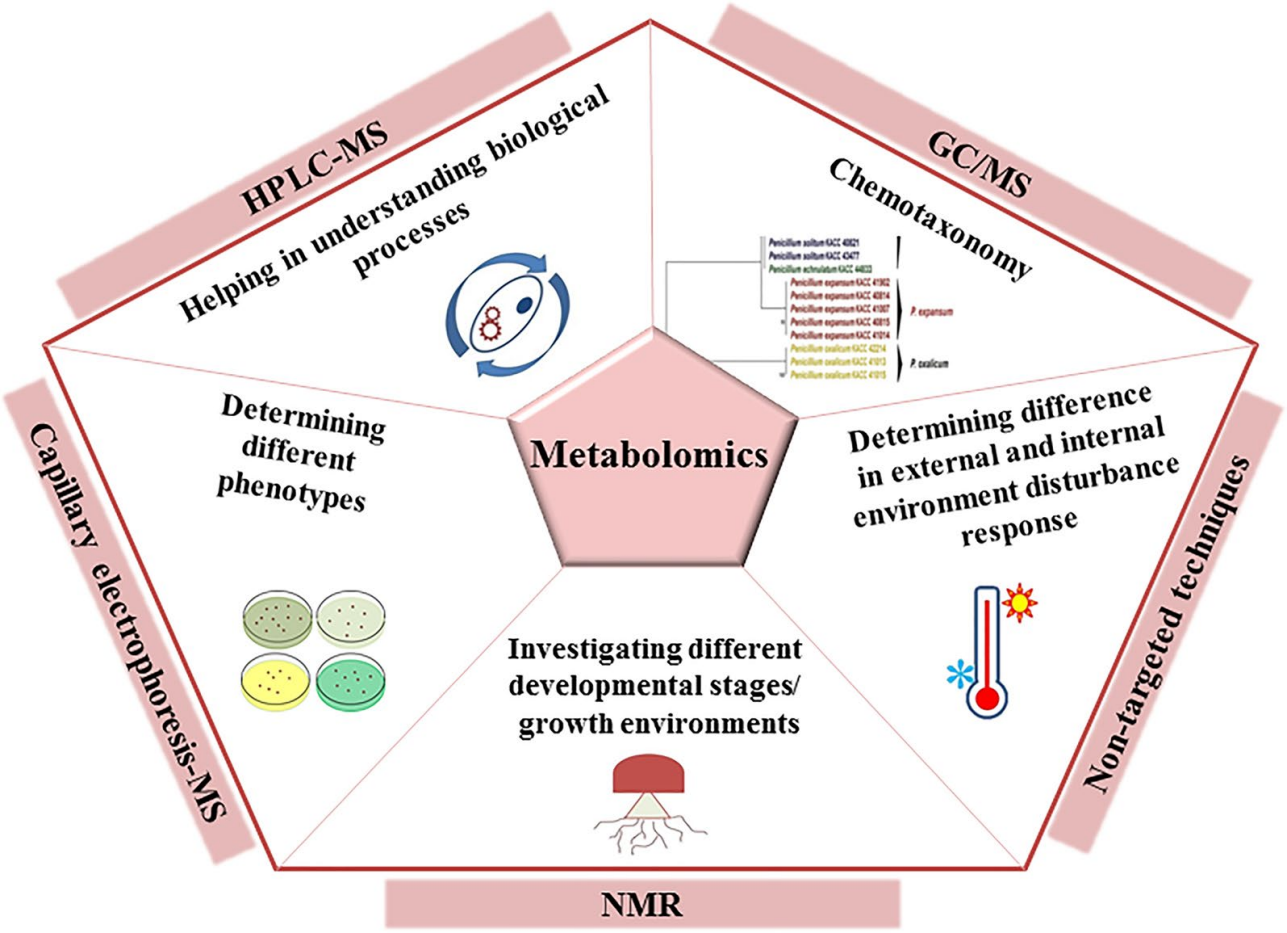
Commonly utilized approaches in metabolomics and a summary of the metabolomics applications in medicinal mushrooms

Ergosterol, along with some of its biosynthetic intermediates, is valuable from an economic point of view, and the products of nearly every stage of ergosterol production are considered drug precursors [233,234]. Wang et al*.* investigated the differences in genes and metabolites in the ergosterol production route throughout the sporocarp development in *F. velutipes* by analyzing the transcriptome and metabolome of samples from three developmental phases. In fact, nine cDNA libraries were obtained from mycelia, young fruiting bodies, and mature fruiting bodies and sequenced via Illumina HiSeq^™^ 4000 platform. A total of 13 DEGs (six up-regulated and seven down-regulated) were identified throughout the development from mycelium to young sporocarps (T1), whereas solely one DEG (one down-regulated) was detected throughout the development from young sporocarps to mature ones (T2). Exploiting nontargeted metabolomics techniques resulted in the identification of a total of seven metabolites (three increased and four reduced) changed in content in the course of T1, and four metabolites were detected to be different in the period of T2. A combined investigation of the genome-wide connection network demonstrated that the metabolites, which were more probable to be adjusted, were chiefly in the post-squalene pathway part of the ergosterol biosynthetic pathway [235]. These results helped in gaining a deeper knowledge of the metabolic route of ergosterol production in *F. velutipes*. Therefore, combining metabolomics data with other omics datasets creates a powerful platform for answering many research questions. In addition to common methods and tools in metabolomics research, a summary of the applications of metabolomics investigations in medicinal mushrooms is provided in Fig. 4.

## Overproduction strategies based on omics data

Utilizing omics data for the design and employment of overproduction strategies have raised the production of some important bioactive compounds in medicinal mushrooms. Results of these investigations are indicated in Table 5. For instance, it was anticipated that Zn2Cys6 transcript factors (mainly CCM_02568 and CCM_01481 genes) might play an important part in improving cordycepin production. Thus, these genes were overexpressed in *C. militaris* CM10. The overexpressed strains (CM10Tf1/CM10Tf2) were subjected to shake-flask fermentation with L-alanine being added after 5 days and results showed that the highest yield of cordycepin in the fermentation medium (99 mg/L) was about threefold higher compared to the wild type. Moreover, the highest yield of cordycepin in the mycelium of the overexpressed strains was 97 ng/g, which is again 3 times higher than the wild type mycelium [198]. Still, there are reports of higher cordycepin production yields even as high as 8.57 g/L by using non-omics-based strategies [240]. Thus, more attempts should be made for optimizing and boosting omics-based overproduction strategies and approaches. This has happened in the study of Ma et al*.* Based on a constructed GSMM and omics data of *G.lucidum*, they had previously found that the yield of extracellular polysaccharides can be enhanced by the addition of l-phenylalanine to the fermentation medium of this mushroom. Optimizing the concentration of l-phenylalanine for the production of extracellular polysaccharides showed that 0.4 g/L of this amino acid results in the maximum production of 0.79 g/L (45.49% increase). However, further optimization regarding the time of L-phenylalanine addition generated more increase in the production of extracellular polysaccharides and their yield was raised from 0.56 to 0.91 g/L by adding 0.4 g/L of the amino acid at 24 h, leading to a considerable increase of 62.50% [241].

**Table 5:** Results of using omics data for designing overproduction strategies in medicinal mushrooms

Strain	Bioactive metabolite	Production of the bioactive metabolite before using data obtained from omics studies	Production of the bioactive metabolite after using data obtained from omics studies	Employed strategy	References
*C. militaris* with doubled cordycepin production induced by L-alanine	Cordycepin	30.04 mg/L	99.83 mg/L (about a threefold increase)	Overexpressing transcription factors CCM_02568 and CCM_01481 of the Zn2Cys6 transcript factors family	[198]
*C. militaris*	Cordycepin	0.049 ± 0.002 g extracellular cordycepin/g dry cell weight (using glucose as the carbon source)	0.094 ± 0.002 g extracellular cordycepin/g dry cell weight (about a twofold increase)	Using xylose as the carbon source	[195]
*C. militaris*	Cordycepin	0.1090 ± 0.0124 g/L	0.3776 ± 0.0055 g/L (3.5-fold increase)	Designing synthetic media by exploiting a GSMM	[236]
*G.lucidum* MDU-7	Phenolics	0.01 mg/mL Galic acid equivalent	0.04 mg/mL Galic acid equivalent (about a fourfold increase)	Using Cu^2+^ as an inducer	[226]
*G. lucidum*	Individual GAs (GA-Mk, GA-T, GA-Me, and GA-S)	GA-Mk: 5.6 µg/100 mg dry cell weight GA-T: 15 µg/100 mg dry cell weight GA-Me: 22 µg/100 mg dry cell weight GA-S: 42 µg/100 mg dry cell weight	GA-Mk: 16 µg/100 mg dry cell weight (2.8-fold increase) GA-T: 40 µg/100 mg dry cell weight (2.6-fold increase) GA-Me: 43 µg/100 mg dry cell weight (1.9-fold increase) GA-S: 53 µg/100 mg dry cell weight (1.2-fold increase)	Overexpressing the squalene synthase gene	[237]
*G. lucidum*	Intracellular and extracellular polysaccharides	Intracellular polysaccharides: 16.84 mg/ 100 mg dry weight Extracellular polysaccharides: 1.21 g/L	Intracellular polysaccharides: 23.67 mg/ 100 mg dry weight (1.4-fold increase) Extracellular polysaccharides: 1.76 g/L (1.4-fold increase)	Overexpressing the α-phosphoglucomutase gene	[238]
*G. lucidum*	Extracellular polysaccharide	0.56 g/L	0.91 g/L (1.6-fold increase)	Adding phenylalanine to the fermentation medium	[3,239]

## Challenges of omics investigations and possible solutions

**Fig. 5:**
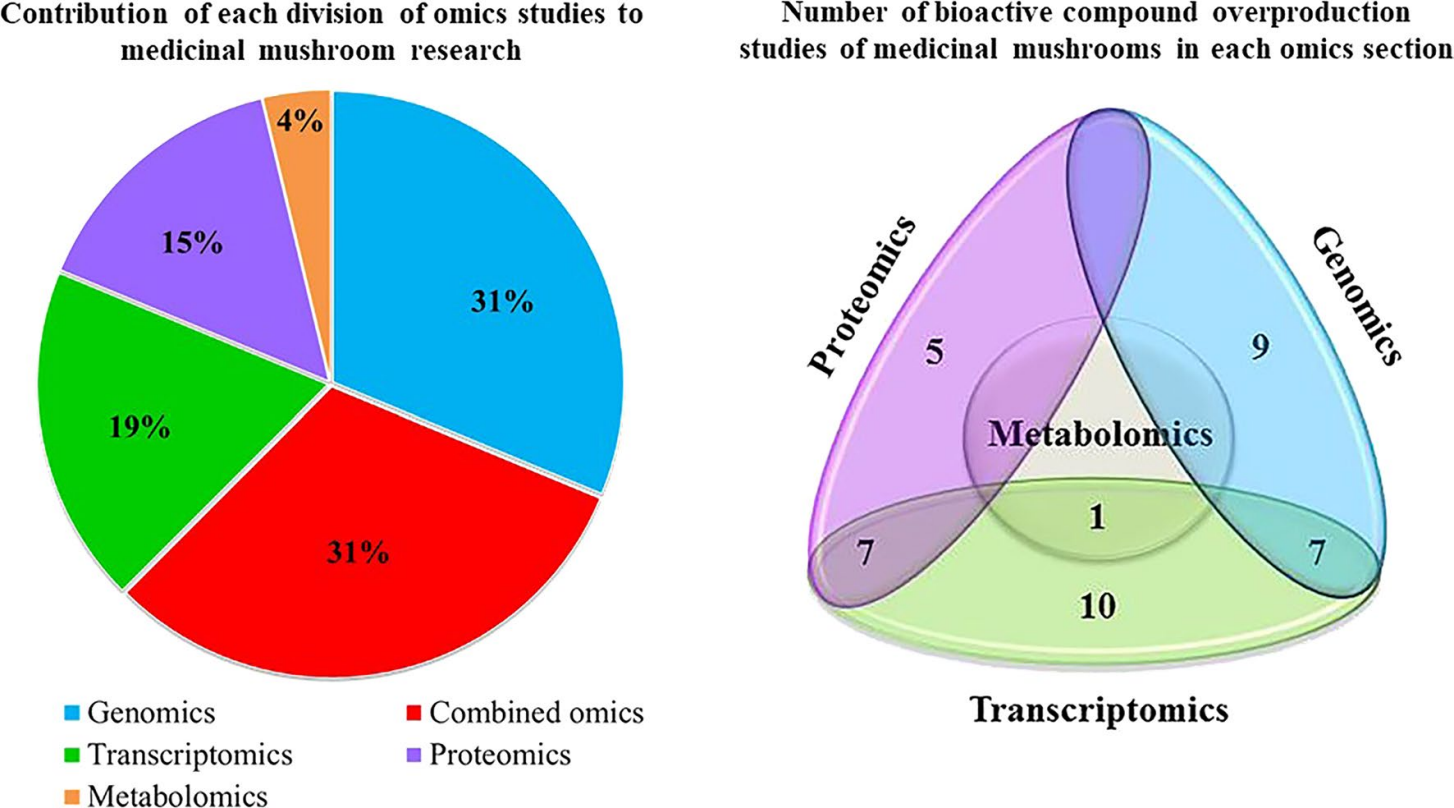
Statistical perspectives of omics investigations in medicinal mushroom research

Based on the studies covered in this review, the statistical contribution of each division of omics studies (i.e., genomics, transcriptomics, etc.) to medicinal mushroom research is demonstrated in a pie chart in Fig. 5. Genomics and integrated omics studies are both considered the most executed omics analyses on medicinal mushrooms. 36% of genomics as well as 57.69% of integrated omics studies produced data that can provide a suitable basis for increasing the production of bioactive compounds in future attempts. According to the pie chart, the next most utilized omics investigation is transcriptomics and 66.6% of the total transcriptomics analyses were allocated to those studies that can facilitate the overproduction of bioactive metabolites. The proteomics studies are in the third rank and 33.3% of these investigations have been performed with the purpose of facilitating the overproduction of the desired metabolites. Finally, metabolomics studies have the least contribution to medicinal mushroom research (4%).

The number of studies associated with bioactive compound overproduction performed in each omics section is also presented in Fig. 5. The number of studies that have utilized more than one division of omics and performed integrated omics investigations is indicated at the intersections. Therefore, most of the omics studies aiming at improving the production of bioactive metabolites are in the field of combined omics, transcriptomics, and genomics, respectively. However, metabolomics and then proteomics investigations have had the least contribution to the overproduction of bioactive metabolites which is possibly due to the limitations and challenges of omics investigations.

For instance, proteome techniques are not meeting expectations, and reaching the complete proteome has not been accomplished yet. As gel-free proteomic techniques hold promise for future proteomics research of edible mushrooms, offer a broader range of protein coverage (such as membrane protein), and allow in-depth screening of protein synthesis and PTMs, designing future omics studies based on these techniques may be advantageous for achieving more comprehensive proteomic data in medicinal mushrooms. On the other hand, processing and analyzing proteomics data (LC/MS and LC–MS/MS data) is a very complicated multistep process which is the main bottleneck for many larger proteomics inquiries. These limitations can be conquered by effective sample preparation, modern mass spectrometry techniques, and extensive data processing and data analysis [252]. Another challenge is that identification of all protein spots cannot be carried out via proteomic analysis, and advancements in the not fully developed proteomics are dependent on experiment expenses and the availability of whole-genome sequences of mushrooms. Finding strategies for lowering the costs can facilitate and accelerate this development.

As de novo transcriptome assembly and analyzing gene expression, even in species with no full genome data, have been facilitated by Illumina sequencing technology, transcriptomics can be assumed to be less dependent on genomic investigations compared with proteomics. Metabolomics studies also face several challenges such as incomplete coverage of metabolites as well as hurdles and expenses in the experimental application, which may explain why they have been conducted to a lesser degree in medicinal mushrooms compared to other omics studies. For example, there are differences in sampling methods, sample preparation, instrumentation, and data mining between laboratories as well as among scientists in the same laboratory. Also, since no single platform is capable of interpreting the complete metabolome due to their specific analytical limitations, it can be hard to decide on the best platform for conducting metabolomic analyses. Still, the choice of analytical platform, which depends on both the sample and the purpose of the experiment, influences the result of the experiment and data recovery [253]. Different methods, which are frequently utilized in omics studies, are compared in Table 6, and a summary of their advantages and limitations is provided.

**Table 6:** Advantages and constraints of common techniques in omics studies

Omics study	Techniques exploited in omics studies	Advantages	Constraints	References
Genomics	Genome mining	•Inexpensive •Can be easily performed in laboratories •Does not need any special skills •Anticipates the chemical structures of bioactive metabolites	•Challenges in formulating the chemical structures •Unable to anticipate the biological activities of bioactive metabolites	[254]
Transcriptomics	RNA-Seq	•No need for probes that are transcript-specific or species-specific •Ability to identify new transcripts, single nucleotide variants, gene fusions, and indels (small insertions and deletions) •Expression quantification across a wider dynamic range (> 10^5^) •Acting more specific and sensitive in contrast with Microarray technology •Easy identification of uncommon and low-abundant transcripts, single-nucleotide polymorphisms, uncommon mutations, formerly unknown gene isoforms, regulatory micro-RNAs, and microbial RNAs	•Computational challenges in the precise annotation of sequences and interpreting information •Effects of biases introduced in the course of cDNA library constitution and sequence alignment on transcript quantitation •Absence of standardization between sequencing platforms and read depth •Considerable start-up costs	[178,179,242–254]
	Microarray technology	•Parallel quantification of thousands of genes from various samples •Easy to use •No need for large-scale DNA sequencing	•Requiring species- or transcript-specific probes •Limited expression measurement at the low and high end by background and signal saturation, respectively •Requiring the physical disruption of cells •Lack of strict standards for data collection, analysis, and validation •Each microarray can only present data about the genes that are included on the array	
Proteomics	2-dimensional gel electrophoresis (2-DE)	•The most powerful and commonly •Used technique for investigating fundamental physiological subjects in fungi •Able to separate several thousand different proteins in one gel •Demonstrating isoforms or post-translational modifications	•Difficulty in achieving membrane, cell wall, and small molecular weight (< 10 kDa) proteins besides very low and very high abundant proteins •Reproducibility concerns •Time-consuming and labor-intensive	[255, 257]
	Difference gel electrophoresis (DIGE) technology	•A gel-based method for relative protein quantification in complex protein samples •Providing more reproducibility and sensitivity over traditional 2D-PAGE gels for differential quantitative investigation of protein expression •Providing a wider dynamic range compared to traditional gel staining •Reducing gel-to-gel variations	•Expensive and difficult to execute •Difficulty in separating hydrophobic proteins •It might solely be utilized when the proteins contain available lysine (for minimal labeling) or cysteine residues (for saturation labeling)	[204,251–253, 257]
	iTRAQ labeling technique combined with two-dimensional liquid chromatography-tandem mass spectrometry (2D LC − MS/MS)	•Powerful analysis of chronological changes of proteomic profiles and investigating candidate genes and signaling pathways related to complex developmental processes of filamentous fungi •High sensitivity and specificity •Beneficial for the identification and quantification of proteins across different isoelectric points and molecular weight ranges, functional categories, cellular locations, and abundances	•Time-consuming •Laborious •Very expensive	[202,254]
	Liquid chromatography combined with mass spectrometry (LC–MS)	•Wide proteome coverage •Suitable accuracy and precision in quantification		[255]
	Gel-free proteomics	•Deeper analysis of complex proteomes by integrating labeled and label-free technologies •In-depth screening of protein synthesis and PTMs •Helping the revelation and determination of proteins (such as low-abundant •Proteins, very high-abundant proteins or proteins with drastic isoelectric points) rarely identifiable in 2DE-based proteome analysis •Utilizing multi-dimensional capillary liquid chromatography combined with tandem mass spectrometry for separation and identification of the peptides attained from the enzymatic digestion of whole protein extracts	•Not able to retain isoelectric point and molecular weight information	[201,206,250]
Metabolomics	Nuclear magnetic resonance (NMR)	•Easy sample preparation •Able to perform metabolite levels quantification •High experimental reproducibility •Possessing nondestructive nature •A suitable platform for large-scale or prolonged clinical metabolomics analyses •Capable of handling different types of samples (liquids, solids, gels) and determining unknown metabolites	•Low sensitivity •Difficulty in standardization of NMR methods and data •Requiring large sample volumes	[253, 256, 258]
	Gas chromatography coupled to mass spectrometry (GC–MS)	•Higher sensitivity compared to NMR •Reproducible retention times •Complete databases for identifying metabolites •Possessing more sensitivity for free fatty acids compared to LC–MS	•Requiring extensive sample preparation •Requiring sample derivitization •The possibility of variation due to sample preparation	[253,256, 258]
	Liquid chromatography combined with single-stage mass spectrometry (LC–MS/UHPLC-MS)	•Short separation time •Easy sample preparation •High resolution •High mass accuracy •Ability to analyze a broader range of metabolites compared to GC–MS •Higher sensitivity compared to NMR	•Possessing destructive nature •Difficult reproducibility of retention times between different systems	[253,256, 258]

At the same time, individual omics investigation technology is faced with obstacles because modulation of cellular activity/metabolism levels has interaction with one another. Hence, it is crucial to use omic technologies integratively to obtain complete data [129]. Thus, although exploiting omics studies in medicinal mushroom research brings about a multitude of benefits, omics technologies are not free of challenges, and since they complement each other, combining omics studies can be beneficial for both achieving improved production of bioactive metabolites and eliminating restrictions.

## Conclusions and future perspectives

There is a growing demand for medicinal mushrooms and their bioactive compounds due to nutritional benefits and pharmaceutical applications. Thus, increasing the production of these bioactive substances is essential for minimizing production expenses and meeting large-scale, commercial, and clinical trial demands. One of the methods which have helped in this regard is the exploitation of omics studies. In this review, the statistical contribution of each division of omics studies to medicinal mushroom research was discussed. The obtained omics data can be viewed as tools and prerequisites for systems biology, metabolic engineering, and cell factory construction endeavors. The cell factories obtained based on omics data will then be able to enhance the validness and rationality of synthetic biology and metabolic engineering approaches. This review highlighted that using omics analyses sets the stage for improving the production of bioactive compounds by discovering the functional genes, enzymes, key metabolic compounds, and biosynthetic pathways associated with their biosynthesis. Facilitating strain improvement, identifying more targets and strategies for metabolic and pathway engineering, establishing networks of metabolism reassignment toward bioactive metabolites, and creating powerful platforms for answering subsequent research questions were other assistive roles of omics in medicinal mushroom metabolite overproduction. Also, according to the quantitative data comparisons made among published investigations, it was demonstrated that creating overproduction strategies based on omics data can cause bioactive metabolite production values to experience increase ranging from 1.2 to fourfold. However, exploiting omics technologies and data for designing overproduction strategies in medicinal mushrooms is still far from sufficient.

Combining different levels of omics analyses and developing tools for genetic engineering facilitates the elucidation of the mechanisms of bioactive compound biosynthesis by higher fungi including medicinal mushrooms. This can eventually result in the overproduction and commercialization of the desired medicinal compounds. Moreover, combining omics data provides a comprehensive and systematic outlook, beneficial for the rational design and formulation of future overproduction strategies. Thus, aside from the need for a deeper focus on omics studies and the integration of their resulting data, future attempts must concentrate on improving these investigations and eliminating their limitations through different strategies. For example, combining the obtained data from omics studies with systems biology technologies such as GSMMs can provide better conditions for ideally designing and optimizing the cultivation mediums and increasing the yield of bioactive substances. It is important to mention that integrating proteomics, transcriptomics, and metabolomics data for gaining a better understanding of cellular biology is considered an obstacle in functional genomics and systems biology. Hence, resolving these issues in omics technologies can be noticeably helpful in improving the production of bioactive compounds. Also, as whole-genome sequences of these mushrooms continue to become accessible, we can expect progress in the field of omics studies, especially proteomics, in the future.

## Data Availability

All data are included in the manuscript and Additional information, and further queries about sharing data can be directed to the corresponding author.

## Abbreviations

BGC: Biosynthetic gene cluster
KEGG: Kyoto encyclopedia of genes and genomes
CCM: Central carbon metabolism
TCA: Tricarboxylic acid
GT: Ganoderic triterpenoid
NMR: Nuclear magnetic resonance
GSMMs: Genome-scale metabolic models
ESTs: Expressed sequence tags
WGCNA: Weighted gene co-expression network analysis
